# Proactive and Reactive Response Inhibition across the Lifespan

**DOI:** 10.1371/journal.pone.0140383

**Published:** 2015-10-21

**Authors:** Peter Smittenaar, Robb B. Rutledge, Peter Zeidman, Rick A. Adams, Harriet Brown, Glyn Lewis, Raymond J. Dolan

**Affiliations:** 1 Wellcome Trust Centre for Neuroimaging, Institute of Neurology, University College London. London, WC1N 3BG, United Kingdom; 2 Division of Psychiatry, University College London, Charles Bell House, 67–73 Riding House Street, London, W1W 7EJ, United Kingdom; 3 Institute of Cognitive Neuroscience, University College London, 17 Queen Square, London, WC1N 3BG, United Kingdom; 4 Max Planck University College London Centre for Computational Psychiatry and Ageing Research, London, WC1B 5EH, United Kingdom; Brown University, UNITED STATES

## Abstract

One expression of executive control involves proactive preparation for future events, and this contrasts with stimulus driven reactive control exerted in response to events. Here we describe findings from a response inhibition task, delivered using a smartphone-based platform, that allowed us to index proactive and reactive inhibitory self-control in a large community sample (n = 12,496). Change in stop-signal reaction time (SSRT) when participants are provided with advance information about an upcoming trial, compared to when they are not, provides a measure of proactive control while SSRT in the absence of advance information provides a measure of reactive control. Both forms of control rely on overlapping frontostriatal pathways known to deteriorate in healthy aging, an age-related decline that occurs at an accelerated rate in men compared to women. Here we ask whether these patterns of age-related decline are reflected in similar changes in proactive and reactive inhibitory control across the lifespan. As predicted, we observed a decline in reactive control with natural aging, with a greater rate of decline in men compared to women (~10 ms versus ~8 ms per decade of adult life). Surprisingly, the benefit of preparation, i.e. proactive control, did not change over the lifespan and women showed superior proactive control at all ages compared to men. Our results suggest that reactive and proactive inhibitory control partially rely on distinct neural substrates that are differentially sensitive to age-related change.

## Introduction

We frequently need to exert rapid reactive control over our actions, such as stopping the car when an animal unexpectedly jumps on to the road. It is also the case that we can avail of informative cues to implement proactive control [1–3], such as when keeping one’s foot close to the brake after passing a warning sign for animals on the road. It has been suggested that proactive control is more ecologically relevant than reactive control for understanding both everyday behavior and impulse control disorders [3,4]. However, the dominant paradigm in the inhibition literature—the stop-signal task—only measures reactive control [5,6]. This task has provided a detailed understanding of how corticostriatal loops and the hyperdirect pathway subserve reactive control [7,8] and how age-related decline in these pathways is associated with impaired reactive control [9]. In contrast, proactive control over actions has only more recently become the subject of studies, in humans as well as rodents [10]. Its neural instantiation may be similar to reactive control, engaging the right inferior frontal gyrus, supplementary motor area and striatum [11–14]. However, other regions seem uniquely involved in proactive control, such as the dorsolateral prefrontal cortex [15]. Here we asked—given the similarity of their neural substrates—whether reactive and proactive control decline at a similar rate across the lifespan. Age-related volume reductions are particularly pronounced in frontal regions and occur at a more rapid rate in men than women [16–19]. We therefore hypothesized that both reactive and proactive inhibitory control would decline with age and this decline would be more pronounced in men than women.

To acquire a large and comprehensive sample we collected data using The Great Brain Experiment, a smartphone app with experiments presented as games [20]. Users of the app also gave their educational attainment and, in a subset of players, a measure of depressive symptoms. Although depression is not thought to be related to reactive inhibitory control [21–23], these previous studies have relatively small numbers of participants and additionally did not test whether depressive symptoms related to proactive control. Prior to testing any of the above hypothesis, we established the reliability of response inhibition measures acquired through smartphones by verifying that assumptions of the race model underlying the calculation of stop-signal reaction time (SSRT) hold for data collected remotely [6,24,25].

## Methods

### Participants

All participants were recruited through The Great Brain Experiment (www.thegreatbrainexperiment.com [20]), a smartphone application (app) that is freely available for download. Between March 11^th^ 2012 and April 3^rd^ 2014 a total of 29,740 participants of at least 18 years old submitted 71,981 datasets (‘blocks’) for the game “Am I Impulsive?” Participants provided informed consent upon starting the app for the first time through a button on the screen. Participants were asked for their age (<18, 18–24, 25–29, 30–39, 40–49, 50–51, 60–69, 70+ years old), gender (male or female), location, education (GCSE, approximately equivalent to US GED or high school through age 16; A-level, approximately equivalent to US high school diploma; university degree; post-graduate qualification), life satisfaction (0–10 in steps of 1). Players were only known to us through an anonymous number. Participants that indicated to be under 18 years old were excluded from the experiment, though they could still play the games. Participants could play the app anywhere they chose to, meaning the experimental environment was left uncontrolled. Ethical approval for this experiment and the consent procedure was obtained from the UCL Research Ethics Committee (4354/001).

### Task

**Fig 1:**
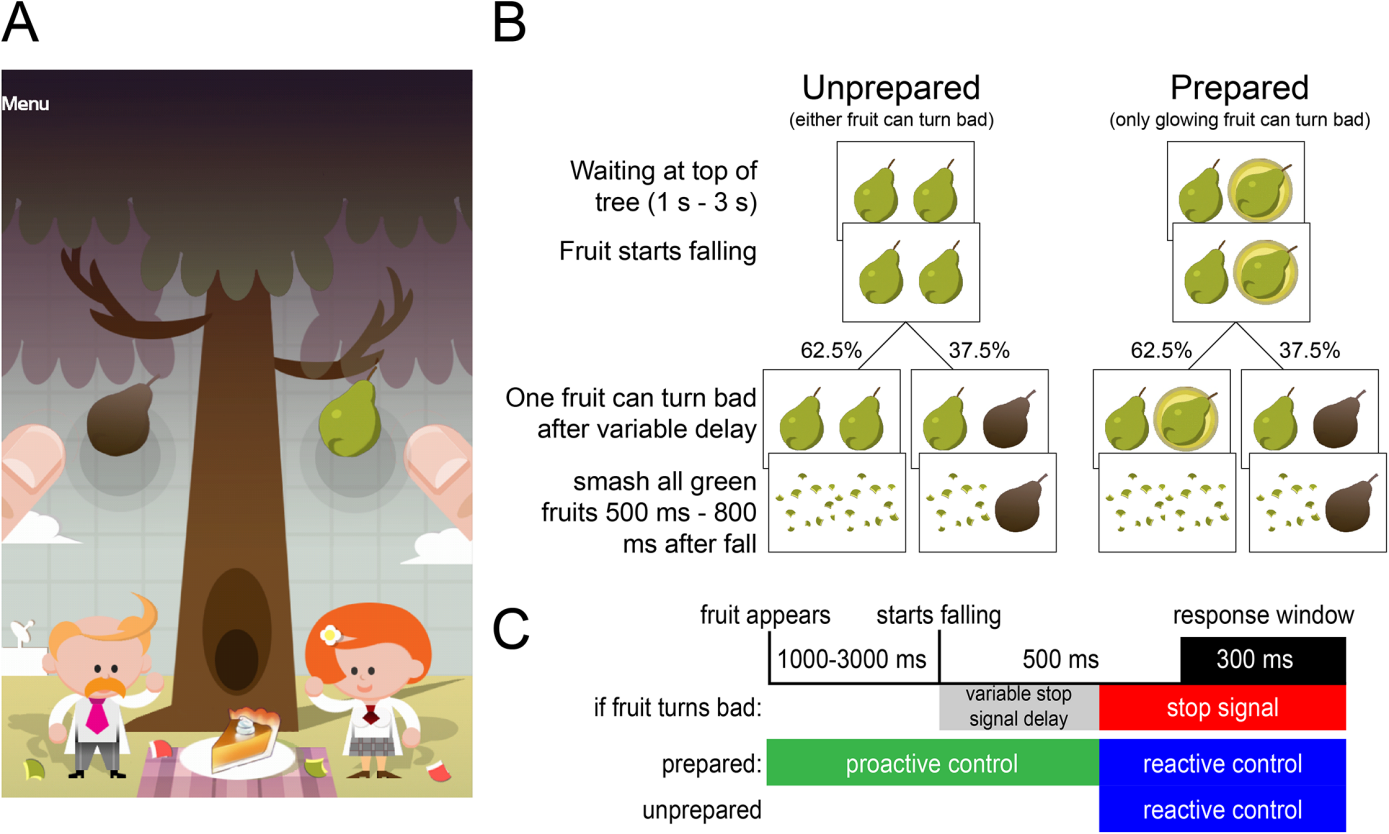
Task design. (A) The game required participants to smash fruits as they passed over the gray circles. (B) On 37.5% of trials one of the fruits turned brown in mid-flight, prompting the participant to quickly withhold their response only on that side. On Unprepared trials either fruit could turn bad (the example in B shows the fruit on the right turning bad). On Prepared trials one of the fruits glowed, indicating that only that fruit could turn bad. (C) This information could be used to employ proactive control, and we quantified proactive control as the improvement in performance in Prepared over Unprepared trials.

The design of the task draws from various existing tasks [12,15,26–28]. Specifically, the task here is a simple response task with two responses and no decision component, where we manipulate the amount of information available to the participant as to which of the two responses might require inhibition. Participants tapped the left and right side of their smartphone or tablet screen to smash two falling fruits (Fig 1A). A single trial consisted of the fruits hanging at the top of the screen for 1–3 s (uniformly distributed), followed by the fruits falling down the vertical axis of the screen. When these passed over two circles indicating the response window, spanning 500 to 800 ms following onset of the fall, subjects were required to tap both sides of the screen (Fig 1B). Out of 32 trials in a single play of the game, a random draw of 12 trials (37.5%) were ‘selective stop trials’ on which one of the fruits turned brown (as if it were rotten), indicating the corresponding side of the screen should not be tapped. On 16 out of 32 trials (‘Prepared’ condition) a glowing circle around one of the fruits indicated to the participant that this fruit alone might turn brown (which it would do in 6 out of 16 trials, i.e. 37.5%). On the other 16 trials (‘Unprepared’ condition) neither fruit glowed meaning either fruit might turn bad. The stop trials were always ‘selective’, i.e. both fruits never turned bad in the same trial. Prepared trials thus contained extra information concerning the action that might require stopping, allowing the participant to prepare and exert proactive control (Fig 1C). The number of milliseconds between the start of the response window and the fruit turning brown is the stop signal delay (SSD). We used separate staircases for the SSD in Prepared and Unprepared stop trials. The staircases started at -200 ms, moved by 50 ms up or down following correct or incorrect stops, respectively, and were reset at each play of 32 trials.

In summary, there were 4 types of trials: Unprepared go, Unprepared stop, Prepared go, and Prepared stop. Reactive control was calculated as the SSRT in the Unprepared condition, whereas proactive control was calculated as the *difference* in SSRT between Unprepared and Prepared trials (i.e. the improvement in SSRT with information [12]). All trials types were fully counterbalanced over events on the left and right side of the screen. We do not report our analyses split out over left and right responses or stop signals, because we have no specific hypotheses regarding left-right differences. The order of trials was randomized for each play. Every play was preceded by a short instruction screen.

### Participant exclusion

Only completed blocks that were immediately sent to our servers over an active internet connection were stored. We first discarded data from participants with no correct Go or Stop trials, no failed or successful stop trials in either Prepared or Unprepared trials, or an SSRT that was smaller than or equal to 0 (see below for SSRT estimation). This left 22,098 out of a total of 29,740 participants (74%). Unless noted otherwise, we performed all analyses on subjects that submitted 2 blocks or more to allow for reliable estimation of the SSRT (12,496 out of 22,098 participants played 2+ blocks, 57% [25]). Data collected over multiple blocks by a single subject were concatenated.

### Beck Depression Inventory (BDI)

A link to an online version of the BDI-2 [29,30] was sent to all participants. In addition to a standard set of 21 questions we asked 5 questions about depression history, number of depressive episodes, duration of depression over lifespan, history of anti-depressant medications, and occurrence of depression in immediate family. In case of duplicate submissions only the first submitted questionnaire was analyzed. In our sample of participants (>1 play) the response rate was 4% (509 participants).

### Estimation of SSRT

The SSRT quantifies the number of milliseconds it takes on average to inhibit an ongoing response after a stop signal appears on the screen. For example, an SSRT of 200 ms would indicate that if a stop-signal were presented 150 ms before a response is going to be made, the subject would not be able to stop and would make an error of commission (referred to here as a ‘stopFail’ trial); if the stop-signal were presented 250 ms before the response, the stop process would finish before the response is executed, thus leading to a successful stop trial (‘stopSuccess’ trial). The SSRT can be calculated for the Unprepared and Prepared condition separately. In this study, SSRT-Unprepared is our measure of reactive control, and the percentage improvement between Unprepared and Prepared, i.e. 100*(SSRT-Prepared—SSRT-Unprepared)/SSRT-Unprepared, is our measure of proactive control.

We computed the SSRT for the Prepared and Unprepared condition separately using the integration method [24,31]. We ordered all leading Go reaction times (RTs)—defined as the time of the first recorded response in the trial in ms from the start of the fall—in descending order and selected the RT corresponding to a participant’s probability of successfully stopping over all stop trials was selected. For example, if the participant successfully stopped their response in 5 out of 12 stop trials, their p(stop) would be 42%. We would then select the RT 42% down the ordered list of RTs (e.g. 640 ms). From this value we subtracted the mean time at which the fruit turned bad relative to onset of the start of the fall (e.g. 350 ms) to obtain the SSRT (in this example, 640–350 = 290 ms). We excluded 18 subjects whose proactive control was larger than 100% or smaller than -100% as outliers.

### Estimation of selectivity

Some studies have shown that inhibition of one response also slows down inhibition of concurrent responses (e.g. [27]). We estimated the selectivity of inhibition by calculating RT_stopSuccess_—RT_Go_, where RT_stopSuccess_ is the response time of the action that was not stopped on successful Stop trials However, our reliability analysis (see Results) showed that this measure cannot be estimated accurately from our data, so in the remainder of the paper we focus on SSRT rather than selectivity.

### Statistical analysis

We regressed the various dependent variables against models that always included nuisance variables including the number of submitted blocks, proportion of correct Go trials (both location and timing of press), and operating system (iOS or Android) in R [32,33]. As regressors of interest we used, across multiple models, scalar variables of age and BDI (both mean-centered; each participant’s age set to the center of their age bin), and factors gender and education. For analyses that included education we excluded participants 18–24 years old as a large proportion might not yet have finished their education. We used the R-package ‘doBy’ for post-hoc contrasts [34]. For reliability analysis and plotting we used MatLab R2012a (The MathWorks, Inc.). We assessed reliability as the intraclass correlation coefficient 1–1 (ICC) as implemented in a custom MatLab script (http://uk.mathworks.com/matlabcentral/fileexchange/22099-intraclass-correlation-coefficient—icc-). The ICC takes into account the degree of absolute agreement between values, such that for normalized data the ICC is equivalent to the correlation, whereas for non-normalized data (as used here) the ICC drops as absolute values deviate from one another. To calculate split-half reliability we compared even to odd blocks (as in [35]). Note that we report effect sizes and 95% confidence intervals in ms rather than p-values, as the large sample size makes p-values less informative [36]. We also focus on the strength of effects rather than statistical significance. Nevertheless, a significant effect at a false positive rate of 0.05 can be concluded if the 95% confidence interval does not cross 0.

## Results

### Basic descriptors of behavior

Table 1 presents basic measures of behavior split out across age groups for comparison with other studies. For the benefit of the reader we further unpack leading Go RT (the fastest response on a trial) as a function of condition, age and gender in S1A Fig; and the variance in leading vs non-leading Go RT (‘non-leading’ refers to the second response in a trial) in S1B and S1C Fig. Note that the average SSRT is in the range of 340 to 400 ms, higher than the average values reported in the literature. We will come back to this point in the Discussion.

**Table 1:** Results on various measures of the task across age groups. ‘Leading’ refers to the RT of the fastest response on each trial, and non-leading refers to the second response on the trial. For definitions of other terms see the Methods. All values are presented as mean with 95% CI over participants unless noted otherwise.

		RT relative to start of response window (ms)				prob. successful stop		prob. correct go		Selectivity (ms)		SSRT (ms)	
		Unprepared		Prepared									
Age (n included)	mean # blocks submitted	Leading	Non-leading	Leading	Non-leading	Unprepared	Prepared	Unprepared	Prepared	Unprepared	Prepared	Unprepared	Prepared
18–24 (2939)	3.4	154 (1.4)	190 (1.8)	155 (1.5)	214 (2.0)	0.64 (0.005)	0.67 (0.005)	0.87 (0.005)	0.81 (0.006)	58 (2.3)	34 (2.2)	365 (2.1)	340 (2.3)
25–29 (2367)	3.7	153 (1.5)	192 (2.1)	155 (1.6)	217 (2.2)	0.65 (0.006)	0.68 (0.005)	0.86 (0.006)	0.80 (0.007)	61 (2.2)	35 (2.4)	365 (2.2)	335 (2.4)
30–39 (3554)	3.9	151 (1.2)	193 (1.8)	154 (1.4)	218 (1.9)	0.63 (0.005)	0.67 (0.004)	0.85 (0.005)	0.78 (0.007)	61 (2.0)	35 (2.0)	373 (1.8)	342 (2.2)
40–49 (2041)	4.5	153 (1.6)	198 (2.4)	156 (1.7)	221 (2.6)	0.62 (0.006)	0.68 (0.006)	0.84 (0.008)	0.75 (0.010)	65 (2.6)	33 (2.8)	384 (2.3)	349 (3.0)
50–59 (1010)	5.8	156 (2.4)	211 (4.3)	157 (2.7)	232 (4.6)	0.61 (0.010)	0.68 (0.009)	0.80 (0.014)	0.68 (0.016)	66 (3.8)	43 (3.9)	399 (3.7)	353 (4.5)
60+ (585)	6.4	151 (3.4)	212 (5.8)	151 (3.9)	229 (6.8)	0.59 (0.014)	0.66 (0.014)	0.73 (0.018)	0.62 (0.022)	60 (6.6)	43 (6.6)	411 (5.5)	365 (6.9)

### Reliability of SSRT and selectivity estimates

**Fig 2:**
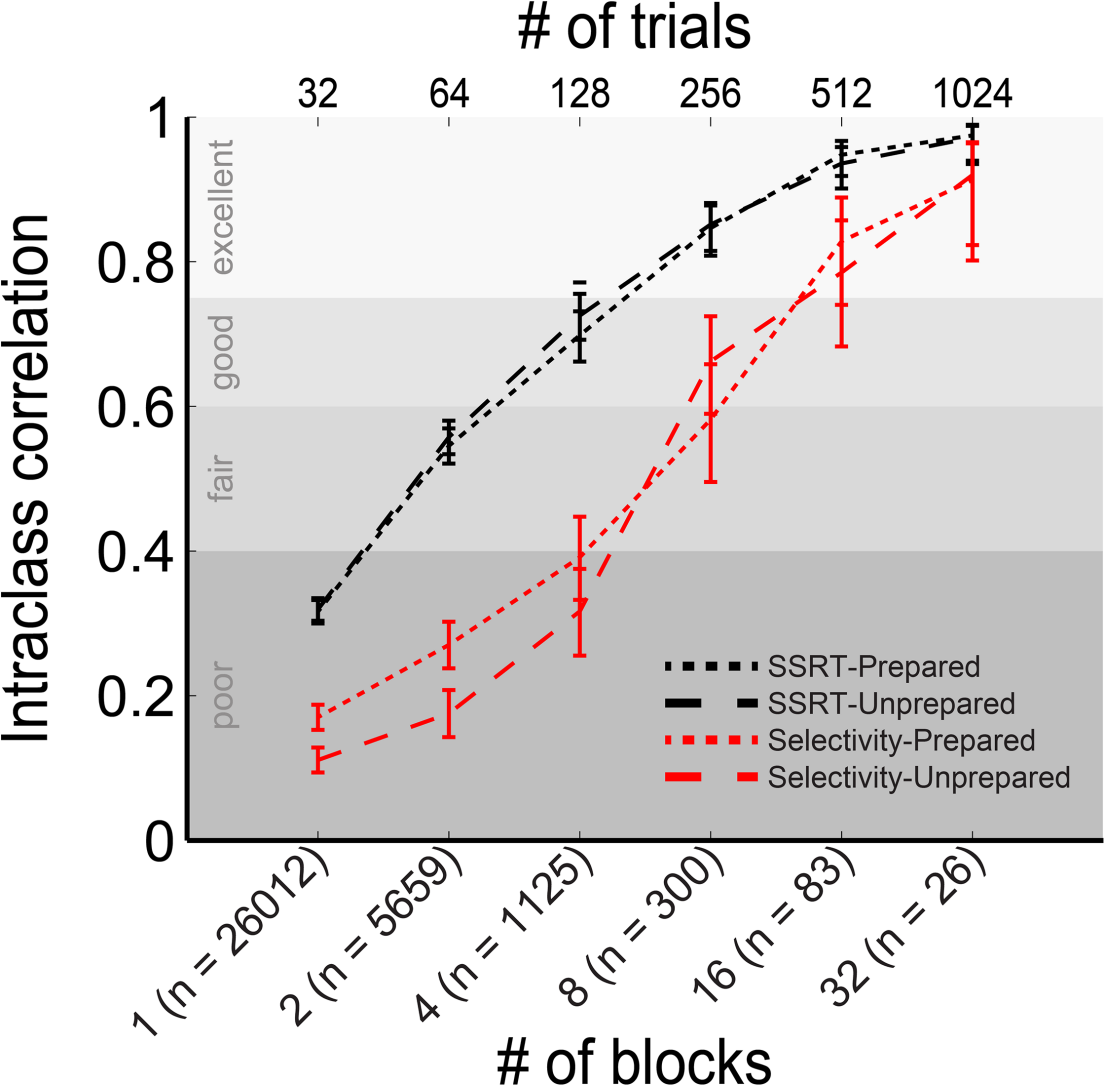
Reliability of SSRT and selectivity depends on the number of available blocks per subject. The SSRT represents the duration of the inhibitory process, i.e. the speed of inhibition. Selectivity represents the slowing that occurs on the concurrent response when an action is inhibited. Here we use the intraclass correlation (ICC) to quantify the reliability of these measures as a function of the number of trials that are used in the estimation and the trial type. If SSRT and selectivity are estimated from the first game only and compared to the second game (# of blocks = 1 in the figure), reliability is ‘poor’ for all measures (following criteria from [37]). Reliability increases as more blocks are used for estimation. However, reliable estimation of selectivity requires approximately 4 times as much data as reliable estimation of SSRT as shown by the rightward shift of the selectivity curves compared to SSRT curves. Error bars represent 95% CI on the ICC, n = number of subjects for whom sufficient blocks were available, e.g. 26,012 participants had at least 2 submitted blocks so that the reliability of the first game to the second game could be calculated.

SSRT and selectivity have been used as markers of inhibitory control (e.g. [12,28]). Their reliability depends on the amount of available data for each subject. For example, 52% of subjects submitted only a single play, which is about 2 minutes’ worth of data. Indeed, for subjects who played more than once, the intraclass correlation (ICC) between the first and second play is below 0.4 for both SSRT and selectivity, which is classified as ‘poor’ (see Fig 2 [37]). Including twice the number of trials leads to a ‘good’ reliability for SSRT-Unprepared and SSRT-Prepared, whereas the reliability of the selectivity measures remains poor (Fig 2). Given these results we excluded those subjects who only submitted a single block, and focused our analyses primarily on SSRT in this paper.

### Applicability of the independent horse race model

**Fig 3:**
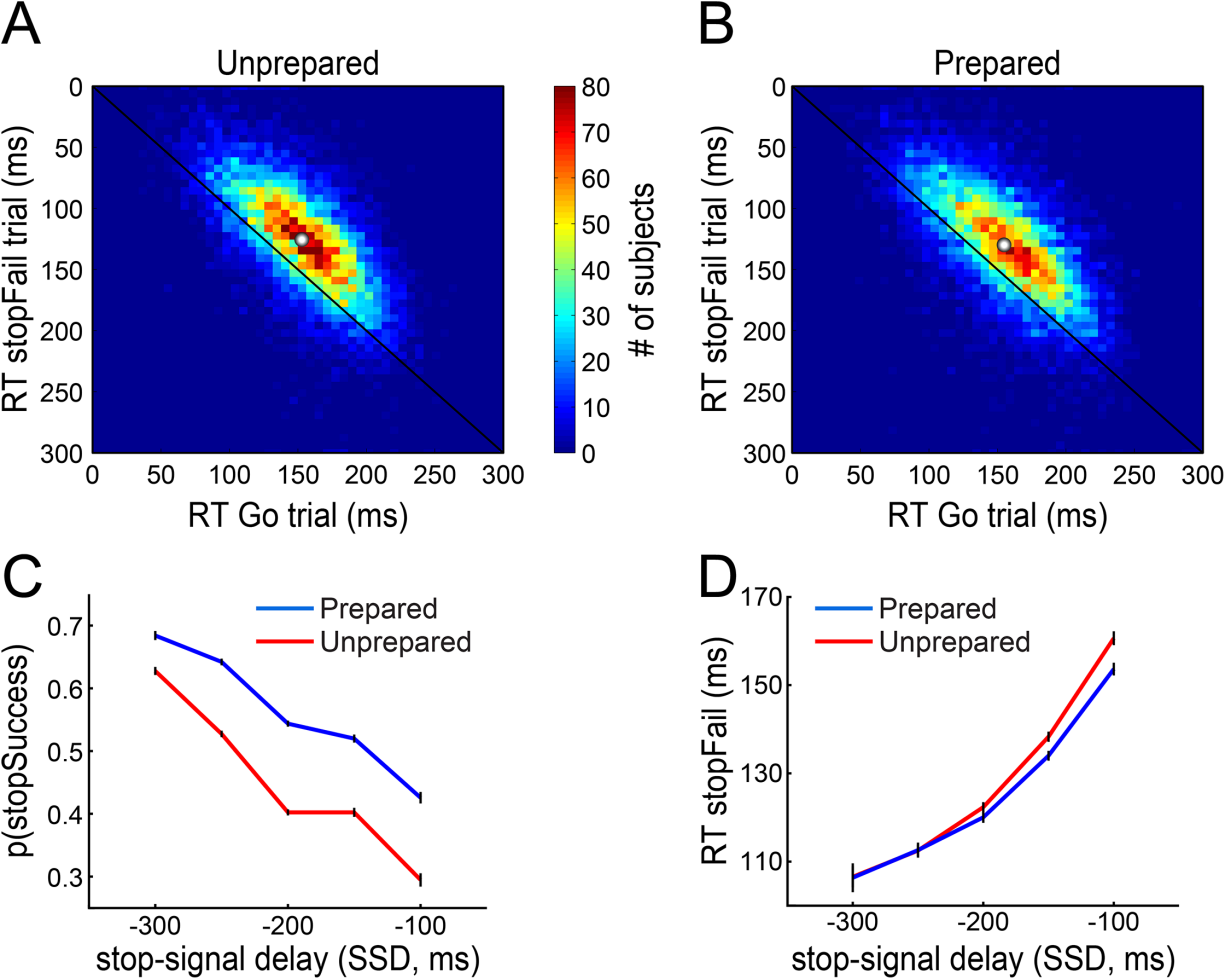
The Unprepared and Prepared condition satisfy assumptions of the race model used to calculate SSRT. All times in this figure are relative to the onset of the response window, which was 500 ms after the fruit started falling. The horse race model [24] assumes that a Stop process with fixed duration is set off as soon as the stop signal is presented. This process then catches up with the Go process only if the stop was initiated far enough in advance of the Go response. This might happen 1) if the Go process happened to be slow on that trial and/or 2) if the stop-signal was presented early. Confirming the first prediction, in stopFail trials (where the subject erroneously responds and thus fails to stop) the reaction times are on average faster than in Go trials, in both the Unprepared (A) and Prepared (B) condition. Confirming the second prediction, the later the stop-signal was presented (i.e. the later the fruit turned brown) the lower the chance of stopping successfully (C). Lastly, if the stop signal is presented close to the onset of the response window then the Stop process cannot catch up even with slow Go responses; this predicts that the average stopFail RT will go up with later stop signals, which is indeed the case (D). Error bars indicate 95% confidence intervals. White dots in A and B indicate population means.

The independent horse race model estimates the speed of inhibition (SSRT) based on both the stop performance and the Go RT distribution. Despite good reliabilities, the relatively small number of trials included in the calculation of some of the SSRTs might make this model unsuitable [24,38]. To ascertain the applicability of the horse race model to our data we checked a range of assumptions of the model (Fig 3; see S2A and S2B Fig for the distribution of the data that goes into the calculation of the SSRT). Firstly, we observed that in trials where the subject failed to stop, the RT tended to be more than 20 ms faster than the average Go RT, in line with the prediction that failed stop trials represent the fast part of the Go RT distribution (Fig 3A and 3B; Prepared: mean RT_Go_—RT_stopFail_ = 22.8 ± 0.6 ms; Unprepared: mean RT_Go_—RT_stopFail_ = 25.3 ± 0.6 ms). Secondly, the later the stop-signal was presented, i.e. the larger the SSD, the lower the probability of stopping (Fig 3C) and the higher the reaction time on failed stop trials (Fig 3D). Together these results suggest the independent horse race model is applicable to data from both the Unprepared and Prepared conditions in subjects with 2 or more submitted blocks.

### Comparison of SSRT to lab data

The SSRT presented here is high compared to values more commonly found in the literature, which are between 200 and 250 ms including in our own research (e.g. [12]). However, lab-based experiments are commonly administered over extended periods of time with ample training, instruction and feedback.

For a fairer comparison to the current results, we estimated the SSRT from the Prepared and Unprepared condition in Smittenaar et al. [12], estimated from the first 100 practice trials. These practice trials were preceded only by 30 trials without any preparation and instruction about the preparation cues similar to that in the app. The mean SSRT was 310 ms in the Unprepared and 330 ms in the Prepared condition, compared to 340 ms and 365 ms, respectively, in 18–24 year olds in the data from the app (note that the Unprepared SSRT being faster than Prepared SSRT can be explained in light of a speed-selectivity trade-off; see [12]). Note these values from the lab practice sessions are approximately 100 ms slower than the SSRTs estimated from the full lab experiment, suggesting an effect of exposure to the task on SSRT. However, we found no evidence for an improvement in SSRT in app players that played multiple blocks (data not shown). This suggests that some of the difference in lab- versus app-based SSRTs might be due to the opportunity for supervised practice in lab experiments. Further implications will be highlighted in the Discussion.

### Preparation improves speed of inhibition

As expected, preparation (proactive control) improved the SSRT (S2C Fig; Unprepared—Prepared, 31.9 ± 1.1 ms improvement). Preparation also improved the selectivity (S2D Fig; Unprepared–Prepared, 26.1 ± 1.3 ms). To examine how our between-subject factors affected inhibitory control we examined SSRT-Unprepared as a measure of reactive control, and the improvement from Unprepared to Prepared as a measure of proactive control.

### Demographics of proactive and reactive control

**Fig 4:**
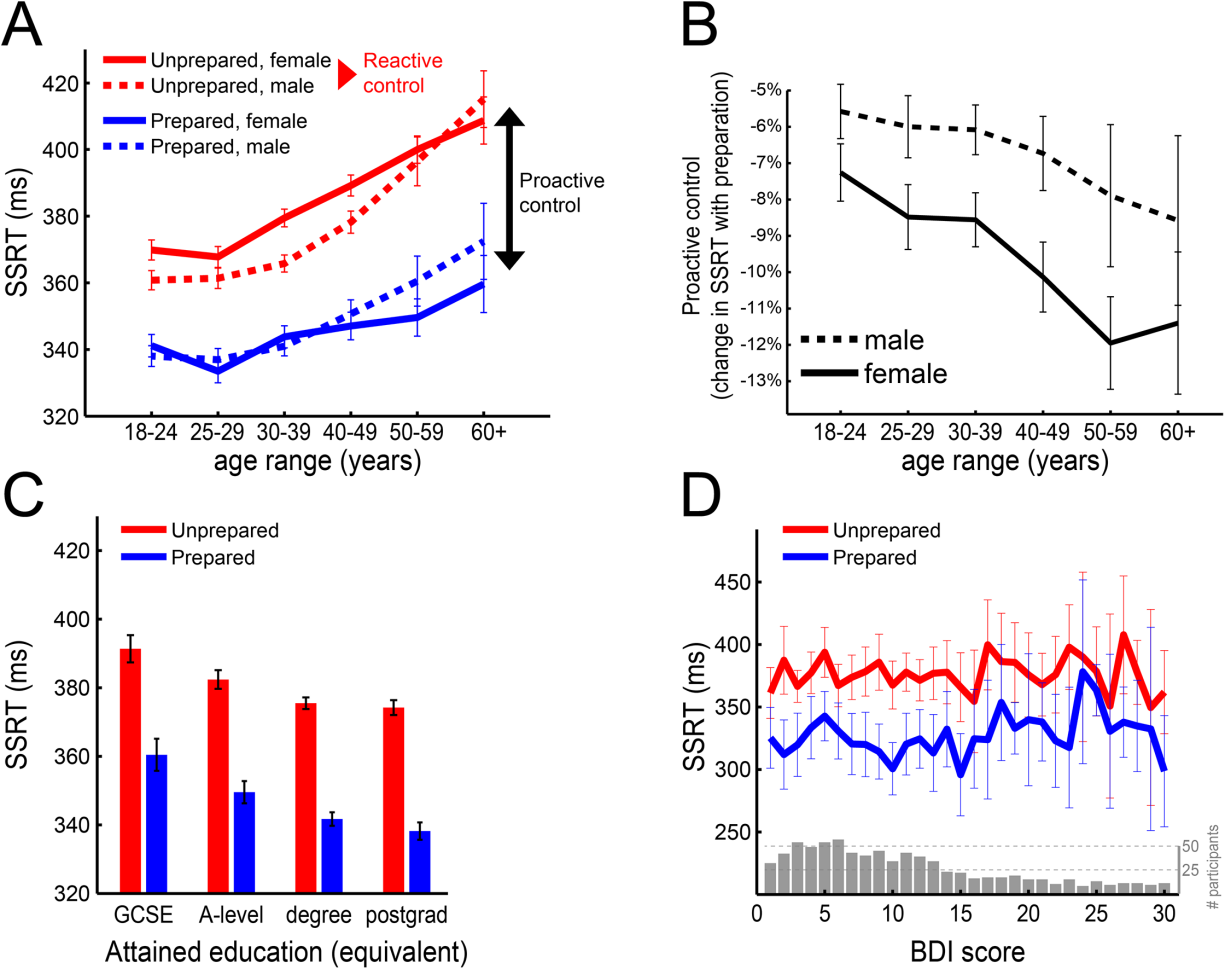
Demographics of proactive and reactive control. (**A**) SSRT-Unprepared, which measures the speed of reactive control, increases with age. However, this age-related decline is more rapid in men than women. Proactive control is quantified as the difference between Unprepared and Prepared SSRT, i.e. the amount by which inhibition is improved through preparation (difference between red and blue line). (**B**) In proactive control we observe a strikingly different pattern. Relative to performance in Unprepared trials, women at all ages improve more with preparation than men. Although the lines have a negative slope, the Age coefficient itself is not different from zero. The y-axis represents the improvement in SSRT between Unprepared and Prepared as a percentage of SSRT-Unprepared, such that more negative values indicate greater benefit of preparation on the speed of inhibition. (**C**) Higher attained education in participants aged 25 or over is not only associated with better reactive control (reduction in SSRT-Unprepared), but also with better proactive control (larger difference between red and blue bar). (**D**) The distribution of BDI scores is shown in the grey histogram. No relationship with reactive or proactive control was apparent. All error bars indicate 95% CI. BDI = Beck Depression Inventory; SSRT = stop-signal reaction time; GCSE = general certificate of secondary education; A-level = general certificate of education advanced level.

In a regression of SSRT-Unprepared on age, gender, age-by-gender and three nuisance variables (see Methods), we observed 18–24 year old women are 9.97 ± 1.99 ms slower than men. However, we also observed a strong age-by-gender interaction on this measure of reactive control (Fig 4A, red lines). That is, whereas men on average deteriorate by 10.1 ± 1.22 ms per decade, women do so only at 8.2 ± 1.05 ms per decade (1.9 ± 1.57 ms per decade slower than men). Reactive inhibitory control thus declines more slowly in women than men.

In a regression with the same six predictors but on proactive control (the difference between Unprepared and Prepared SSRT), 18–24 year old women showed larger improvement with preparation than men (Fig 4B; 1.95 ± 0.57 percentage point (p.p.) difference). This effect of gender on proactive control, if anything, became stronger with increasing age (the gender difference increased by 0.46 ± 0.45 p.p. per decade). Although Fig 4B suggests proactive control increases with age, there was no evidence for a main effect of age (-0.01 ± 0.03 p.p.) due to the inclusion of various other regressors (Table 2). The two regressors attenuating the main effect of age were the age*gender interaction described above as well as the number of correct Go trials. The former was added as we had a priori hypotheses regarding the existence of such an interaction; the latter was included as a proxy for general skill in and comprehension of the task goals (as opposed to specific inhibitory skill).

**Table 2:** Changes in the Age regression coefficient as various regressors are added to the regression model. As suggested by Fig 4B there is a strong main effect of Age when only Age is used as predictor. As this table reveals, the addition of the age*gender interaction and the number of correct Go trials as nuisance regressors diminishes the Age coefficient.

Regressors in model; dependent variable is proactive control (% change in SSRT)	Regression coefficient for Age ± 95% CI
Age	-0.10 ± 0.02 (p < 2e-16)
Age+gender (no interaction)	-0.09 ± 0.02 (p = 2.7e-14)
Age*gender (main effects + interaction)	-0.06 ± 0.03 (p = 0.0005)
Age*gender + operating system	-0.06 ± 0.03 (p = 0.0008)
Age*gender + blocks played	-0.06 ± 0.03 (p = 0.0003)
Age*gender + correct Go trials	-0.02 ± 0.03 (p = 0.20)
Full model: Age*gender + operating system + blocks played + correct Go trials	-0.01 ± 0.03 (p = 0.50)

Together, these results suggest that reactive, but not proactive, control deteriorates with age, but more so in men than women (Fig 4A). Furthermore, women experience greater benefits from proactive control across all ages (Fig 4B).

For education we also examined SSRT-Unprepared and the difference between SSRT-Unprepared and SSRT-Prepared to distinguish reactive versus proactive control (Fig 4C). The predictors were education, age, gender, age*gender and three nuisance variables, with 18–24 year olds excluded. Progressively higher levels of education were associated with better SSRT-Unprepared (compared to GCSE, in ms: A-level, -4.8 ± 4.1; degree, -7.1 ± 3.7; postgrad, -8.4 ± 4.0). Similarly, proactive control increased with education (compared to GCSE, in percentage points: a-level, -1.6 ± 1.2; degree, -2.6 ± 1.1; postgrad, -3.3 ± 1.2). Together, this shows that a higher level of education is associated with better reactive as well as proactive control.

Lastly we explored possible relationships between depression indices and SSRT (Fig 4D). In a regression identical to the age-by-gender regression in Fig 4A, but with BDI scores added as a predictor, we observed no relationship between the BDI score and SSRT-Unprepared (change in SSRT with every point on BDI scale, 0.05 ± 0.39 ms) or proactive control (0.04 ± 0.14 p.p.).

## Discussion

Here we present a dataset acquired through smartphones in which we examined inhibitory control in a large group of male and female participants with a wide range of age, education and depressive symptoms. We found that these data conform to assumptions of the widely used independent horse race model, which was used to estimate the SSRT in the Prepared and Unprepared conditions. These measures can be reliably estimated from only ~4 minutes of data (64 trials). We found that reactive control, operationalized as SSRT in the Unprepared condition, deteriorates with age, and this occurs faster in men than women. Proactive control, operationalized as the change in SSRT in the Prepared compared to the Unprepared condition, was not related to a main effect of age. Moreover, proactive control was stronger in women compared to men at all ages. Additionally, we found higher levels of education are associated with better reactive as well as proactive inhibitory control, whereas depression shows no relationship with either measure of inhibitory self-control.

Our results extend previous research on inhibition in multiple ways. We replicate the finding that reactive control deteriorates with age [9,35,39]. Unlike these previous studies we observed an age-by-gender interaction. This contributes to an ongoing debate on differential cognitive decline in men and women depending on task type, with some reports finding faster decline in men (on a cognitive battery [40]), and others finding no differential decline (in spatial ability [41,42]), and yet others finding women decline faster (on simple and choice RT [43]). This cognitive heterogeneity is surprising given the more rapid age-related neural decline consistently found in men compared to women [16–19,44], including in prefrontal regions critical for inhibitory control [11,12]. Future work, for example on white-matter connectivity rather than brain volume [9], might shed more light on the neural underpinnings of age- and gender-related decline and maintenance of cognitive function. It is particularly important to understand how proactive control performance is maintained in the face of neural decline, and how this aligns with theories of proactive control in aging [2,45].

In contrast to this gender-by-age effect on reactive control, proactive control was greater (i.e. SSRT improved more with preparation) in women compared to men at all ages. That is, whereas advance information benefits the speed of inhibition across age groups and genders, this benefit was greater in women than men at all ages. This suggests that preparatory benefits are not simply a function of baseline performance whereby worse performers show larger improvements. Although another study found that preparation paradoxically slows SSRT [28] or has no effect on SSRT [26], all these results can be explained as a trade-off between speed and selectivity of inhibition [12]. That is, depending on instruction and disposition of the participant, the preparatory cue might be used to improve the speed *or* selectivity of inhibition, with both trading off against one another.

Education is often controlled for (e.g. [46]) or measured but not reported on (e.g. [39,47]) in the response inhibition literature. To the best of our knowledge there have been no previous reports showing education is associated with better reactive and proactive inhibitory control. However, these effects might not be specific, and indeed education was also associated with e.g. differences in performance on Go trials (data not shown). The specific relationship between education, IQ and inhibitory control therefore remains unclear.

We observed strong relationships between demographics and SSRT estimated from these data. Nonetheless, the average SSRT is high compared to previous work studying similar types of inhibition [11,12,28]. An unusual aspect of the task is the fact that a Go response can be anticipated from the falling fruit, compared to having an unpredictable onset in the standard stop-signal task. This feature was inspired by previous work, in which SSRTs were either in the standard 200 to 250 ms range [27,48] or considerably higher ([15],approximately 330 ms; [49]). The latter authors note that more complexity and working memory load can increase SSRT [50,51] and that less intense stop-signals also lengthen SSRTs [52,53]. We also speculate that some of the higher SSRTs might arise from the lack of supervised practice provided to the participant in the app. Indeed, when we re-analyzed data from the initial practice phase in Smittenaar et al. [12] these SSRTs were approximately 100 ms slower than those estimated from the full experiment. Additional factors that might contribute to slower SSRTs in smartphone compared to lab data include the uncontrolled environment which might be more distracting, the small screen size, the heterogeneous participant group compared to university undergraduates, the way the stop signal is presented, the intermittent participation compared to continuous play in the lab, and the minimal instruction.

As such, there are many benefits as well as limitations to the use of smartphones in cognitive science [54]. As in lab-based [55] and online [56] cognitive research here we assume the same sampling bias and additionally the cross-sectional approach could have affected the results (e.g. [57]), much as it does in all cross-sectional aging research. Broad sampling of the wider population is a first step to understanding how our models of cognition apply beyond the common context of Western university students. For example, another game from The Great Brain Experiment showed that a computational model of subjective well-being developed in the lab could be used to predict well-being in the broader population [58]. Smartphones, given their ubiquity not only in the West but worldwide [59], provide a cost-efficient and societally engaging way of achieving this goal.

The dataset presented here is publicly available for further research. Our approach shows the power of harnessing large-scale public participation in psychology and academic research [60]. By transforming laboratory tasks into engaging games [61], researchers can now engage the public in research at vast scales.

## Supporting Information

S1 Fig Description of Go reaction times. The leading Go reaction time is defined as the average time after the start of the response window (which itself starts 500 ms after the fruit starts falling) that the first button is pressed. The second response in the trial is called the non-leading Go RT. (A) This graph illustrates how leading Go RT changes with gender, age and preparation. Participants successfully responded around the center of the response window, which is at 150 ms on this scale. (B-C) By definition, non-leading Go RTs are slower than leading Go RTs. Each point in the plot is a participant, and the red dot indicates the mean of the population. In the Prepared condition the non-leading response was 64.0 ± 1.1 ms slower than the leading response; the equivalent value for Unprepared is 42.5 ± 0.9 ms.
(TIF)

S2 Fig Calculation of SSRT and distribution of SSRT and selectivity. (A-B) These figures illustrate the distribution of 12,496 participants in the space of leading Go RT, SSD and p(stopSuccess) for the Unprepared condition. These three variables together determine the SSRT through the quantile method. That is, a fast SSRT arises from a high average SSD, fast Go RTs and high p(stopSuccess). (C) Selectivity is defined as the speed of the remaining response after a successful stop minus the average leading Go RT, such that larger values indicate worse selectivity of the inhibitory process. Selectivity is better in the Prepared compared to Unprepared condition. The black-white dot indicates the mean of the population. (D) The SSRT, reflecting the speed of inhibition, is faster in the Prepared compared to Unprepared condition.
(TIF)
