# Supplementary figures and images for: Proactive and Reactive Response Inhibition across the Lifespan
Source: PLoS One. 2015 Oct 21;10(10):e0140383. doi: 10.1371/journal.pone.0140383 (PMC4619547; doi:10.1371/journal.pone.0140383)

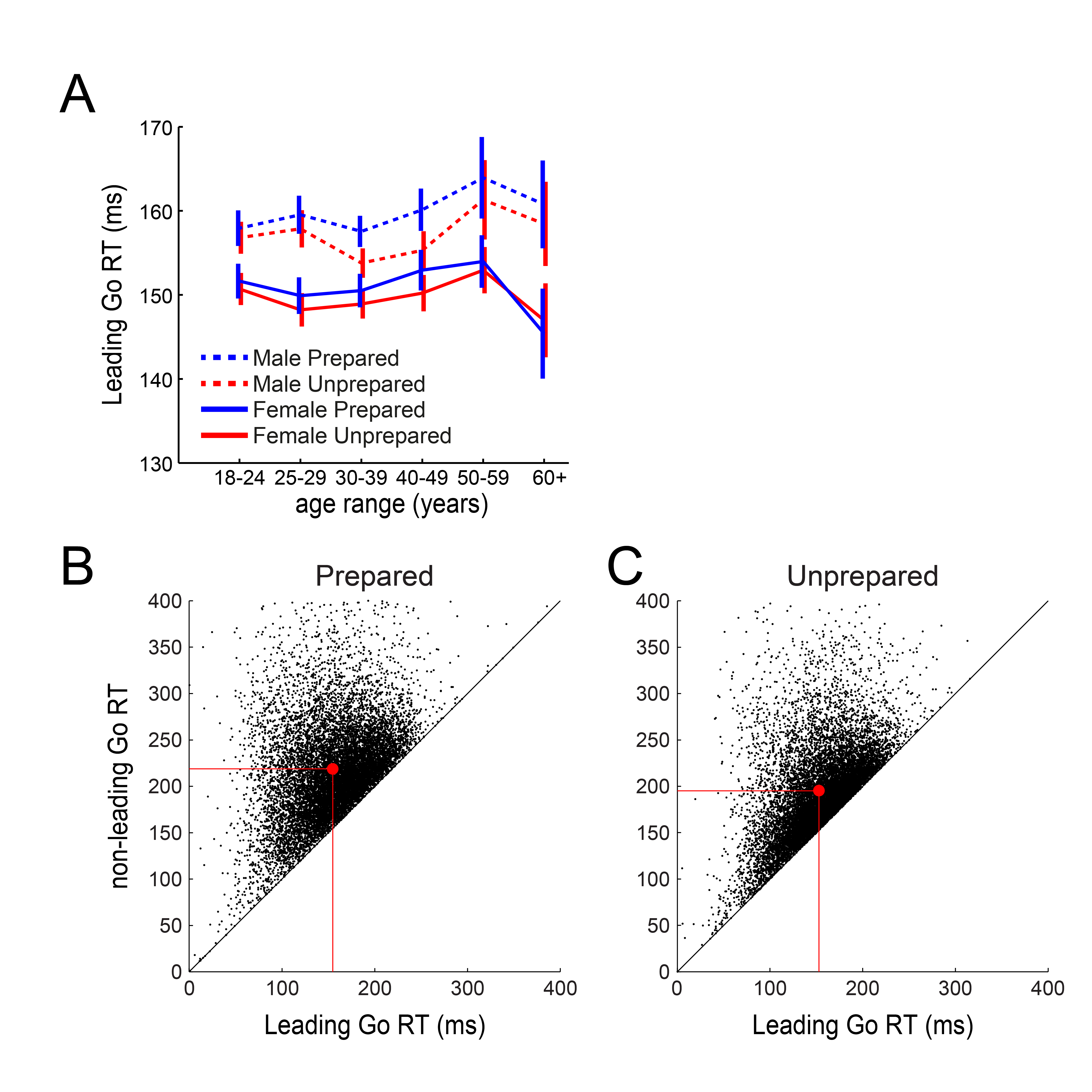

Supplement: S1 Fig — The leading Go reaction time is defined as the average time after the start of the response window (which itself starts 500 ms after the fruit starts falling) that the first button is pressed. The second response in the trial is called the non-leading Go RT. (A) This graph illustrates how leading Go RT changes with gender, age and preparation. Participants successfully responded around the center of the response window, which is at 150 ms on this scale. (B-C) By definition, non-leading Go RTs are slower than leading Go RTs. Each point in the plot is a participant, and the red dot indicates the mean of the population. In the Prepared condition the non-leading response was 64.0 ± 1.1 ms slower than the leading response; the equivalent value for Unprepared is 42.5 ± 0.9 ms. (TIF) [file pone.0140383.s001.tif]

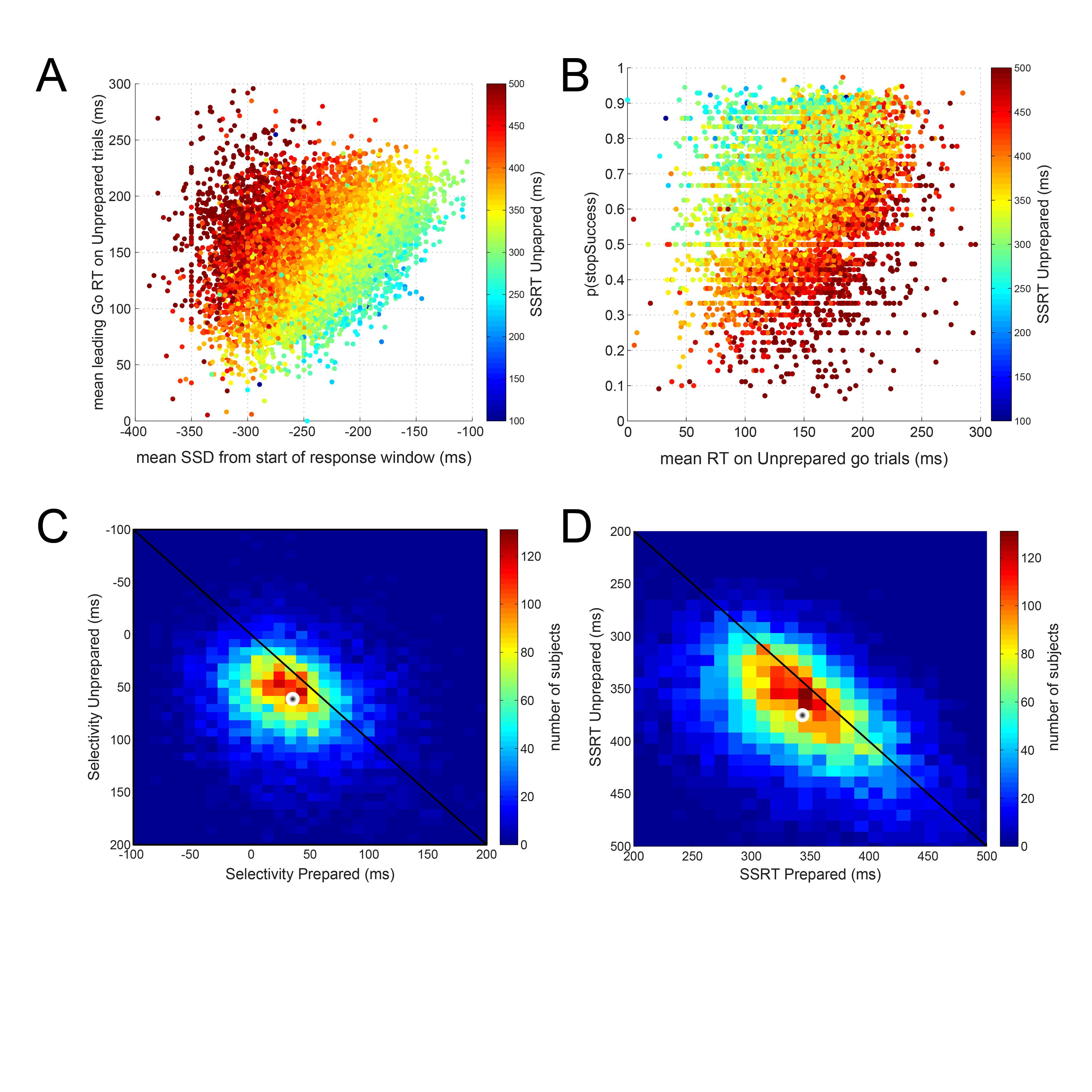

Supplement: S2 Fig — (A-B) These figures illustrate the distribution of 12,496 participants in the space of leading Go RT, SSD and p(stopSuccess) for the Unprepared condition. These three variables together determine the SSRT through the quantile method. That is, a fast SSRT arises from a high average SSD, fast Go RTs and high p(stopSuccess). (C) Selectivity is defined as the speed of the remaining response after a successful stop minus the average leading Go RT, such that larger values indicate worse selectivity of the inhibitory process. Selectivity is better in the Prepared compared to Unprepared condition. The black-white dot indicates the mean of the population. (D) The SSRT, reflecting the speed of inhibition, is faster in the Prepared compared to Unprepared condition. (TIF) [file pone.0140383.s002.tif]
